# Diet and Nutraceuticals for Mental and Physical Performance in Athletes

**DOI:** 10.1007/s40279-022-01794-w

**Published:** 2022-11-30

**Authors:** Lawrence L. Spriet

**Affiliations:** Human Health and Nutritional Sciences, University of Guelph, Guelph, ON N1G 2W1 Canada

This supplement stresses the importance of diet and selected nutraceuticals that may impact both the physical and mental performance of athletes. The past 2.5 years living with the coronavirus disease 2019 pandemic have taxed all of us mentally and possibly to a greater extent in the athletic population. While life in general has returned to some form of normalcy for many, emerging from this pandemic has alerted us to the importance of vaccines and physical-distancing measures, especially as they relate to sport. The return of organized sport at all levels has also underscored the important roles that exercise and sport play in the lives and mental health of everyone, including athletes. The goal of this supplement is to provide recent information that will help athletes achieve optimal physical and mental performance in their chosen sport.

The Gatorade Sports Science Institute has been bringing sports nutrition and sports science researchers together for ~ 37 years to discuss many topics that relate to the nutrition, performance, and well-being of athletes. Since 2012, these meetings have been known as the Gatorade Sports Science Institute Expert Panel. The worldwide coronavirus disease 2019 pandemic necessitated that the latest meeting in October of 2021 was again held in a virtual format. However, the meeting was a great success and following the meeting, the authors summarized the recent work in their topic area, resulting in the papers in this *Sports Medicine* supplement (the ninth in a series supported by the Gatorade Sports Science Institute).

The first paper in this supplement examines new horizons in the application of carbohydrate research for endurance athletes [1]. Despite the immense knowledge that exists regarding the importance of carbohydrates as a fuel for high-intensity endurance activities, recent advances regarding the recommended type and quantity of carbohydrates to be ingested before, during, and after intense exercise bouts have been made. In addition, periodizing carbohydrate intake based on many factors, including the goal and demand of training or competition, suggests that a much more personalized approach to carbohydrate recommendations is needed [1]. Finally, the knowledge gained from emerging technological advances such as continuous glucose monitoring is likely to further fine tune carbohydrate dietary recommendations. The second paper in this supplement examines the immense interest in ingesting ketone supplements in athletic settings [2]. The recent commercial availability of ingestible exogenous ketone supplements in the form of ketone salts and ketone esters, and other ketogenic compounds, results in an acute transient increase in circulating acetoacetate and β-hydroxybutyrate levels, which has been termed “acute nutritional ketosis” or “intermittent exogenous ketosis”. While a few studies suggested that exogenous ketone supplements may improve endurance performance, recovery, and over-reaching, most studies have failed to observe benefits of acute nutritional ketosis on performance or recovery. It is also clear that these exogenous ketone supplements are not a viable fuel for athletes engaging in intense exercise [2].

The third paper examines the potential effects of caffeine and co-consumed active ingredients on improving and/or maximizing mental performance in sport [3]. Caffeine is a plant defence compound that is consumed to improve performance in sporting contexts, with potential benefits in both physiological and psychological domains. Caffeine does modestly and consistently improve alertness and fatigue. Its effects on mental performance are restricted to improving attention or concentration with no effects on working memory, executive function, and long-term memory. Caffeine also interacts with many bioactive medicinal and dietary compounds, potentially modulating the time course of their functional effects and providing benefits to mental performance that exceed those associated with caffeine alone [3]. However, future research needs to isolate the relative contributions of the component parts of these products that contain several potential active ingredients.

The fourth paper addresses the latest research examining whether there may be some benefit from ingesting dietary-derived nicotinamide adenine dinucleotide (NAD^+^) precursors, which may increase the NAD^+^ content in skeletal muscle where it is a vital energy intermediate [4]. Nicotinamide riboside and nicotinamide mononucleotide supplements have been shown to display a health benefit in humans with clinical NAD^+^ deficiencies. However, the animal and human nicotinamide riboside supplementation studies to date suggest that NAD^+^ therapeutics do not alter skeletal muscle metabolism or improve athletic performance in healthy humans [4].

It is encouraging to see that researchers are now aware of the large need for more research examining active female individuals in general and also female athletes. The fifth paper in this supplement addresses the fact that women are the largest consumers of dietary supplements, and that supplements can play a role in the health and athletic performance of women over the life span [5]. While more female nutrition and exercise-specific research is needed, existing data and the physiological differences between the sexes support new product development and evidence-based education for active women regarding the use of dietary supplements. The authors suggest that the areas where nutritional ingredients may help support the health and well-being of active women include body composition, energy/fatigue, mental health, and physical health [5].

The final paper in this supplement addresses the athlete gut microbiome, which has received a tremendous amount of interest of late [6]. The human gut microbiome is a complex ecosystem of microorganisms, that is of the utmost importance to human health. Many studies have reported differences between the gut microbiomes of athletes and nonathletes, with health-associated bacteria often positively associated with physical activity. Other studies have suggested that the microbiome composition may play a role in the intense exercise-induced gastrointestinal and respiratory infections that athletes experience. An exciting prospect for many elite athletes and sports nutrition personnel is that athletic performance and health may some day be improved via microbiome manipulation [6]. While research of the gut microbiome is increasing, the relationship between exercise and the microbiome remains under-investigated and is a fruitful area of work for sports nutrition investigators.

The papers of this supplement have summarized the recent advances in areas related to diet and nutraceutical supplementation that may positively impact the mental and physical well-being and performance of athletes. As always, more research is needed and ongoing in these important areas of sports nutrition. We encourage the readers of these papers to disseminate the present knowledge related to these topics and generate new knowledge to help answer the many remaining questions.
